# Multi-Wavelength Biometric Acquisition System Utilizing Finger Vasculature NIR Imaging

**DOI:** 10.3390/s23041981

**Published:** 2023-02-10

**Authors:** Jerzy Fiolka, Krzysztof Bernacki, Alejandro Farah, Adam Popowicz

**Affiliations:** 1 Faculty of Automatic Control, Electronics and Computer Science, Silesian University of Technology, Akademicka 16, 44-100 Gliwice, Poland; 2 Instituto de Astronomía, Universidad Nacional Autónoma de México (UNAM), Ciudad Universitaria, Mexico City 04510, Mexico

**Keywords:** biometry, finger vasculature, image processing

## Abstract

Personal identification using analysis of the internal and external characteristics of the human finger is currently an intensively developed topic. The work in this field concerns new methods of feature extraction and image analysis, mainly using modern artificial intelligence algorithms. However, the quality of the data and the way in which it is obtained determines equally the effectiveness of identification. In this article, we present a novel device for extracting vision data from the internal as well as external structures of the human finger. We use spatially selective backlight consisting of NIR diodes of three wavelengths. The fast image acquisition allows for insight into the pulse waveform. Thanks to the external illuminator, images of the skin folds of the finger are acquired as well. This rich collection of images is expected to significantly enhance identification capabilities using existing and future classic and AI-based computer vision techniques. Sample data from our device, before and after data processing, have been shared in a publicly available database.

## 1. Introduction

The issue of restricted access to resources, only for authorised persons, is a rapidly growing area of technology. This applies to access to confidential resources, but also to access to bank accounts, safe-deposit boxes, and other places where access is to be restricted and only available for specific persons. Nowadays, modern security solutions are applied in several stages, i.e., you need to have something (e.g., a card, hardware token, mobile phone or other electronic device with Internet access), then you need to know something (e.g., the pin code, password, sign, or symbol). A selected biometric feature is additionally used as a further verification step [1,2].

Biometric features can be divided into two groups: (1) behavioural, which can include, for example, handwritten signatures, manner of walking or characteristic eye movements, voice, keystroke dynamics, etc., and (2) physiological which include, for example, fingerprint lines, hand geometries, facial feature geometries, voice colour, ear geometry, iris and retinal characteristics, vascular system of the fingers of the hand, and many more [3]. This essentially makes the biometric system function a pattern recognition system.

An authentication system is susceptible to attacks where attempts to manipulate the data may occur as a consequence. One solution to improve the security of biometric systems is to use several biometrics which is called a multimodal approach [4]. When two or more biometric features are used, the security and effectiveness of the biometric system can be enhanced [3,5].

For the selection of biometric features, it should be taken into account that the feature solution should be characterised by:

- Reliability—the solution must generate minimal false detections;
- Uniqueness—no two people should have the same set of characteristics;
- Acceptability—no user objections to measurement/use;
- Reasonable cost of the solution.

For solutions using a single biometric trait, one of the most intensively developed areas is the use of the vascular system of the fingers of a human hand (FVS, finger vascular system) [6]. In this case, the use of a special apparatus is necessary for data acquisition: an illuminator and a camera/sensor for near-infrared imaging have to be utilized at a minimum. An additional very important property of the vascular system is its invisibility to outsiders and often assumed a priori long-term stability [7,8,9]. However, the former has not not been clearly confirmed. Especially, over a period of tens of years, the invariability of the vascular system may seem questionable because visible changes in the geometry of the vascular system may occur as a result of existing diseases. In the article [10], the authors present what the vascular system looks like in patients with various diseases, e.g., vibration disease, or in a patient requiring blood dialysis. By comparing the images, it can be deduced that after longer periods, the vascular system indeed can change so that the image identification/classification algorithms should be re-trained from time to time.

Some of the FVS-related publications concern instrumental issues, e.g., the selection of an appropriate NIR length [11,12], the assembly of an appropriate combination of lengths used to improve identification performance [13], or novel acquisition devices [12,14,15,16,17]. The algorithmic issues at the pre- and post-processing stages are considered as well [12,18]. The development of the new data processing methods is very intensive [19,20] and is now mostly concerned with the use of neural networks, machine learning, advanced mathematical functions, and other solutions.

A natural, highly efficient identification solution using the FVS seems to be to create a 3D image of it. However, this is a rather complicated process requiring considerable computing resources, a more complex mechanical part of the system (e.g., using moving parts), requiring usually more space and, in many cases, more than one camera. This increases the cost of the device making it less attractive commercially and also, through considerable expansion, less reliable when compared to fixed systems equipped with a single camera [21,22,23].

Table 1 collects information on the devices and systems used to acquire images of the vascular system of the fingers, as described in scientific publications. The table shows both publicly available databases with images taken by the described devices as well as only device concepts. After reviewing the described databases with the images presented in Table 1, it can be seen that there is no database or device that allows for the collection of many biometric features at once.

This paper proposes a more efficient and robust finger biometric feature acquisition system. The presented device allows for an increase in the number of biometric features while maintaining the simplicity of design (no moving parts, single detector/camera), thus ensuring high reliability and low manufacturing cost. Detection of blood pulse reduces the possibility of using an artificial hand as an artefact for unauthorised access to resources [24]. The proposed solution allows the following features to be analysed: (1) the vascular system obtained by illumination with NIR diodes of three lengths and a total of twelve discrete illuminator positions, (2) the external texture of the finger due to the external illuminator used, (3) the shape of the finger, (4) the shape of the rim, and (5) the pulse wave. The proposed solution, currently being tested as a prototype, can be successfully miniaturised and, thanks to its simplicity and lack of mechanical components, brought to the level of a reliable and compact commercial device.

**Table 1:** Databases of known FVS biometric databases/devices.

Name	Vol.	Imgs.	NIR Length	[1]	[2]	[3]	[4]	[5]	[6]	[7]	[8]	[9]
**SNU** [25] (2009)	20	200	830	XB	X	-	-	-	-	-	-	X
**THU-FVFDT** [26] (2009)	60	120	890	XB	X	-	-	-	X	-	-	-
**PKU** [27] (2010)	5208	50,700	850	XB	-	-	-	-	-	-	-	X
**SDUMLA** [28] (2011)	106	3616	890	XB	-	-	X	-	-	-	-	-
**HKPU** [29] (2012)	156	6264	850	XB	X	X	-	-	-	-	-	X
**UTFVP** [30] (2013)	60	1440	850	XB	-	-	-	-	-	-	-	-
**MMCBNU** [31] (2013)	100	6000	850	XB	X	-	-	-	-	-	-	-
**CFVD** [32] (2013)	13	1345	850	XB	X	X	-	-	-	-	-	-
**VERA** [33] (2014)	110	440	850	XB	-	-	-	-	-	-	-	-
**FV-USM** [34] (2014)	123	5904	850	XB	X	-	-	-	-	-	-	X
**GUC-FPFV** [35] (2014)	41	1500	870	XB	X	X	X	-	-	-	-	X
**GustoDB** [11] (2017)	107	11,556	730, 808, 850,	XTB	X	-	-	-	X	-	-	X
			860, 875, 880,									
			890, 940, 950									
**PMMDB** [36] (2018)	47	188	850, 950	XT	X	-	-	X	X	-	-	-
**PLUSVein** [12] (2018)	60	3600	808, 850, 950	XTB	X	-	-	X	X	-	-	X
**SCUT-SFVD** [37] (2018)	100	3600	850	XB	X	-	-	-	-	-	-	-
**MPFVS** [38,39] (2018)	63	252	808	XTB	X	-	-	-	X	-	X	X
**3DFM** [40] (2020)	66	132	850	XTB	X	X	X	-	X	-	X	X
**3FVFKP** [41] (2021)	203	8526	850	XTB	X	-	-	-	-	-	X	-
**Proposed** (2022)	-	-	730, 875, 940	XT	X	X	-	X	X	X	X	X

[1]: vascular system; [2]: finger shape; [3]: finger texture; [4]: fingerprint; [5]: lunula shape; [6]: dermal folds; [7]: pulse; [8]: rotation/image at different angles; [9]: finger vascular profile. X: biometry possible to acquire; B: vascular system of the underside of the fingers; T: vascular system of the upper part of a finger.

## 2. Overview

**Figure 1:**
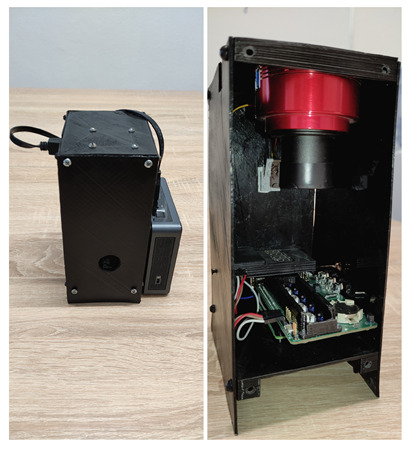
The designed acquisition unit in the ready-to-use version (**left**) and its interior after removing the front panel (**right**). On the back panel, there is an LED illuminating the upper surface of the finger (on the figure this element is partially covered by the camera).

The purpose of this study was to design and fabricate a multi-wavelength biometric acquisition system used for finger vasculature NIR imaging. Figure 1 shows the device before and after removing the front panel. The Intel NUC mini-PC used only to collect images from the camera is connected on the left side image.

Important at the stage of prototyping new concepts is the right choice of hardware platform. Considering its huge popularity and high computing capabilities, the system is based on the Raspberry Pi (RPi) platform. The RPi has the advantage of a huge community, very good quality technical documentation, and the availability of programming libraries in many languages (e.g., Python, C, C++). This choice has decisively facilitated the integration and testing of individual parts of the device. The abundance of publicly available libraries to support existing components or peripherals attached to the RPi, allowed us to quickly test several variants and choose the right solution. The availability of WiFi and Bluetooth wireless connectivity, an Ethernet port, and USB or HDMI connectors are also worth mentioning. Moreover, RPi users can choose from several variants of operating systems based on Linux, Unix, or Windows.

All these features not only ease the development stage, but can also provide an excellent starting point for creating a stand-alone version. In this case, a camera (using a USB or CSI camera port) and touchscreen (using DSI display port) can be attached to the RPi, creating a low-cost stand-alone device. The high computing power of the RPi, as well as the availability of software tools for image processing, such as OpenCV, Python libraries (e.g., Scikit-image, PIL/pillow, NumPy) will significantly facilitate the development of custom processing algorithms.

**Figure 2:**
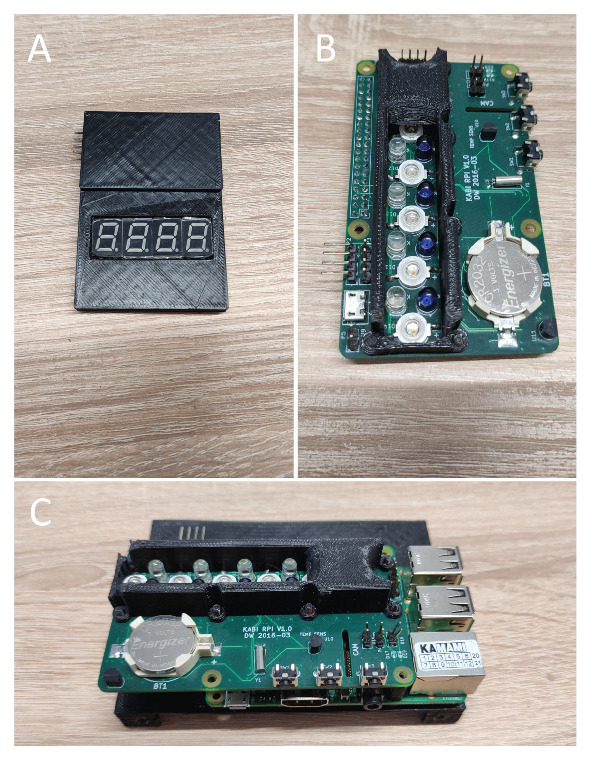
Internal parts of the acquisition device: (**A**) 7-segment module with its driver and in the 3D printed case; (**B**) our designed and fabricated module with NIR LEDs; (**C**) the NIR LEDs module on top of the Raspberry Pi.

Implementation of the basic functionality, which is the ability to back-illuminate the finger with NIR LEDs, required the design and fabrication of a dedicated electronic circuit attached to the RPi via a standard GPIO connector (see Figure 2B,C). In addition to the components, a special housing was placed on the PCB to provide a finger rest. Inside, there are 15 NIR LEDs, arranged in 3 rows of 5 each (in the conducted tests, we were limited to 12 LEDs—3 × 4, as this provided full illumination of the finger). To control the diodes, a specialized multi-channel LED controller was used to which the MOSFET transistors were connected to extend the range of currents flowing through the diodes.

A ZWO ASI178MM camera (monochrome, with a resolution of 3096 × 2080 pixels) equipped with a CMOS sensor, attached via a USB 3.0 interface to the workstation, was used to capture images. The camera has a 2.5 mm wide-angle CS lens (imaging angle of 170 deg.) with a mounted IR filter that cuts out a portion of the spectrum (longpass filter model FEL0650 by Thorlabs, Cut-On Wavelength 650 nm [42]). By cutting off the visible region, potential stray light coming from outside can be reduced. Our imaging device used a pixel-binning mode (called binning 2 × 2) and narrowed the imaging area to a longitudinal slice 260 pixels wide. This allowed us to increase the capture rate to ∼130 frames per second.

A unique feature of the device is the ability to acquire images very fast. This was dictated by a need to reduce the time an individual keeps the finger in our device, as well by the wish to observe pulse waveform in all of the diode utilized. A natural way to acquire finger images for different combinations of NIR diode activation seemed to be to use a simple mechanism in which the workstation sends a request to light the appropriate combination of diodes. When the request is fulfilled, the device sends an acknowledgment, which initiates the start of camera image capture. Unfortunately, tests carried out at the initial stage of the research showed that this method does not achieve a satisfactory speed of image acquisition. This is due to the fact that sending an exposure request to the camera is accompanied each time by sending its full configuration. In the case of the camera used, this allows it to obtain a maximum of a dozen images per second, which was too small a number when wishing to record the pulse wave under illumination by each of the 12 diodes.

Assuming an example heart rate of 90 bpm and having 12 diodes, it can be calculated that the minimum signal sampling rate, according to the Nyquist criterion, should be about 36 samples per second. The previously cited values indicate that using the discussed single-shot approach does not meet this requirement. In the present work, this problem was solved by using Free-Run (FR) mode in which the camera’s configuration parameters are transferred only at the beginning of the acquisition. Unfortunately, once recording begins, there is no synchronization between the backlight diode module and the camera frames (data from the camera is continuously stored in a RAM buffer, which can be accessed only after imaging is completed).

To ensure adequate data synchronization, an additional set of 7-segment displays was used, attached to the RPi via a port expander (see Figure 2A). Since the finger obscures the NIR LEDs during image acquisition (it is impossible to estimate their state), by placing the 7-segment displays in the field of view of the camera, the current pattern of the LEDs’ illumination was easily and clearly integrated into the recorded image (a set of two 7-segment displays would be enough for the present state of 12 diodes, but we found an inexpensive module with 4 displays and were satisfied with it). By using appropriate timing control of both NIR and 7-segment diodes and by using post factum analysis of the resulting image series, it was possible to extract frames for each NIR LED turned on. The details of these two approaches are presented in the *Software* and *Post-processing* sections, respectively.

### 2.1. Hardware

**Figure 3:**
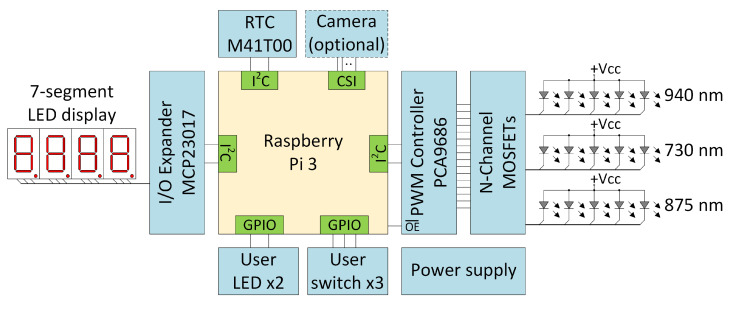
Block diagram of the proposed multi-wavelength biometric acquisition system. NIR LED diodes (940, 730, and 875 nm), 7-segment displays, PWM Controler PCA9686, and MOSFET transistors driver are attached externally. Connection with camera and RTC M41T00 is expected in a future stand-alone version. It is not utilized in the current prototype.

The block diagram of the device is shown in Figure 3. The main component is a single-board computer (SBC—Single Board Computer) Raspberry Pi 3 Model B equipped with 1 GB of RAM, with the Raspberry Pi OS operating system installed. The implementation of the biometric acquisition system required the design and fabrication of a HAT (Hardware on Top) add-on board. The dimensions of the HAT are 56 × 95 mm. The HAT has a connector compatible with the Standard Raspberry Pi 40-pin GPIO header. In the middle part of the add-on board, three rows of LEDs, five elements each, are mounted in a special casing. The diodes placed in successive rows differ in emitted wavelength and optical power:

- 940 nm, 140 mW optical power, L514EIR1B (LIRED5B);
- 875 nm, 210 mW optical power, TSHA5205 (VISHAY);
- 730 nm, 240 mW optical power, OSR9XAE3E1E (OPTOSUPPLY).

The PCA9685 chip, which is a 16-channel, 12-bit PWM Fm+ I2C-bus LED controller, was used to control the NIR LEDs [43]. Each LED output can be on or off or operates with a duty cycle that is adjustable from 0–100 % with 12-bit resolution (4096 steps). The frequency used for PWM control can be adjusted in the range 24–1526 Hz. Due to the need to use currents exceeding the controller’s limit—25 mA—external drivers built with the N-channel enhancement mode MOSFET ($I_{DMAX}$ = 200 mA) [44] were attached to the PCA9685 chip.

Due to the limited number of GPIO lines of the RPi, a module containing 4 7-segment displays was realized using a 16-bit I/O Expander with I2C Interface, MCP23017 [45]. In order to simply and clearly represent the state of the NIR LEDs that illuminate the finger, only 3 segments are used in each of the displays. As can be seen in Figure 3, there are 15 backlight NIR LEDs (3 rows of 5). Importantly, only 12 were used during the conducted tests, as they provided full illumination of the finger. Mechanically, the module is a separate board enclosed in a black case attached via wires to the SDA and SCL lines (and power supply) of the RPi (Figure 2A). Arranging the 7-segment display along the fingertip (i.e., as a longitudinal extension of it) is very advantageous, as an increase in the number of frames acquired from CMOS-type cameras can be achieved only by vertically narrowing the imaging area.

The device also features a simple user interface consisting of three microswitches and two LEDs. One of these LEDs was used to illuminate the upper surface of the finger. The LED used was TSHG5510 with an emitted wavelength of 830 nm and an optical power of 55 mW [46]. The brightness of the diode is determined by a current-limiting resistor, the value of which was chosen experimentally. Considering the possibility of further expansion towards a stand-alone station, the device was additionally equipped with a real-time clock (RTC) with battery backup, M41T00 [47]. However, this functionality was not used in the conducted research.

### 2.2. Software

In Free-Run mode, the camera records data at maximum speed, and the device, independently of the camera, displays sequentially predefined patterns of diode illumination with their representation on 7-segment displays. Taking into account the way of image acquisition in the CMOS sensor (the so-called rolling shutter), it is necessary to maintain certain time dependencies between events, such as switching the NIR LEDs illuminating finger and the LEDs on the 7-segment display. In addition, it is necessary to adjust the length of the time interval in which the camera can obtain a complete and stable image of the finger with the illumination pattern turned on. In addition, the extraction of individual frames from the recorded video imposes restrictions on the time during which both the NIR LEDs and the 7-segment display are off. This time must allow a single dark frame (reference frame) to be recorded, delineating successive combinations of NIR LEDs illumination in the video sequence.

**Figure 4:**
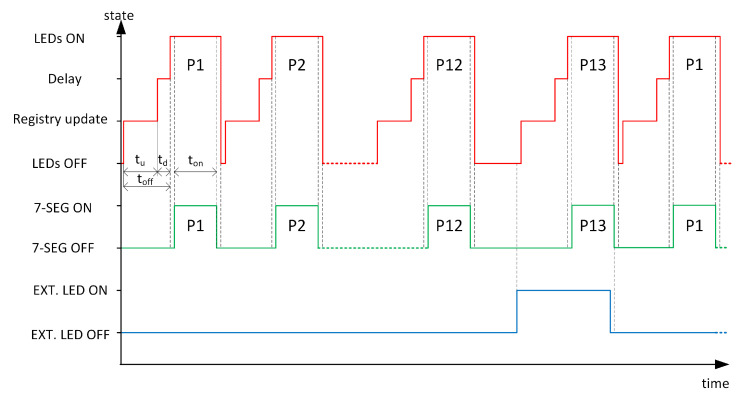
Timing diagrams utilized for control of NIR LEDs, external illumination LED, and the 7-segment display.

Details of the illumination sequence are presented in Figure 4. Let t=0 denote the start of the system. The NIR LEDs, 7-segment displays, and the illuminating diode are then turned off (“LEDs OFF”, “7-SEG OFF”, “EXT.LED OFF”). Then, the procedure for updating the contents of the PCA9685 controller registers (“Registry update”) begins. A Python script, running on the RPi determines the appropriate values, and then this data is sent to the NIR LEDs driver via the I2C interface (the duration of this process depends mainly on the hardware capabilities, i.e., the I2C transmission parameters and how the operating system handles the transmission). This time, $t_{u}$, is determined by hardware, while the duration of the “Delay” state, $t_{delay}$, has been set to a length of $t_{delay}=3$ ms. Thus, we can control the time $t_{off}=t_{u}+t_{d}$ when all NIR LEDs are off (dark frame in the video). Then, the selected NIR LEDs corresponding to the user-defined P1 pattern are turned on (“LEDs ON”), which is accomplished by setting the OE (Output Enable) line of the PCA9655 controller to a low state.

The next step is to switch on the 7-segment display to present the status of the illuminating NIR LEDs. This moment marks the beginning of the period when the frame taken from the camera will contain the image of the illuminated finger along with the correct pattern displayed on the 7-segment displays. The length of the time $t_{on}$ was set to 10 ms. This time is related to the exposure time of the camera, which is 5 ms. By setting the lighting time to 10 ms, we can be sure that at least one exposure in the recorded video will cover the NIR LEDs and 7-segment both turned on. At the end of the $t_{on}$ period, the 7-segment displays are turned off (“7-SEG OFF”), after which all NIR LEDs are turned off at the same time (the OE line of the PCA9685 controller is set high).

The described scheme is repeated many times in order to register images corresponding to subsequent patterns. In the conducted tests, 12 patterns of illumination without the participation of external illumination, P1-P12, were defined. The last pattern, P13, is displayed with external illumination (“EXT.LED ON”).

### 2.3. Post-Processing

Due to the rolling shutter in the camera, individual rows of images are downloaded at slightly different times. This sometimes results in registration of images for which the NIR diodes were switched off or on during the exposure. In order to extract the final images, i.e., those in which the finger is fully illuminated by a selected NIR diode, we apply an appropriate post-processing algorithm (see Algorithm 1).
**Algorithm 1:** Post-processing algorithm for extraction of final image samples.
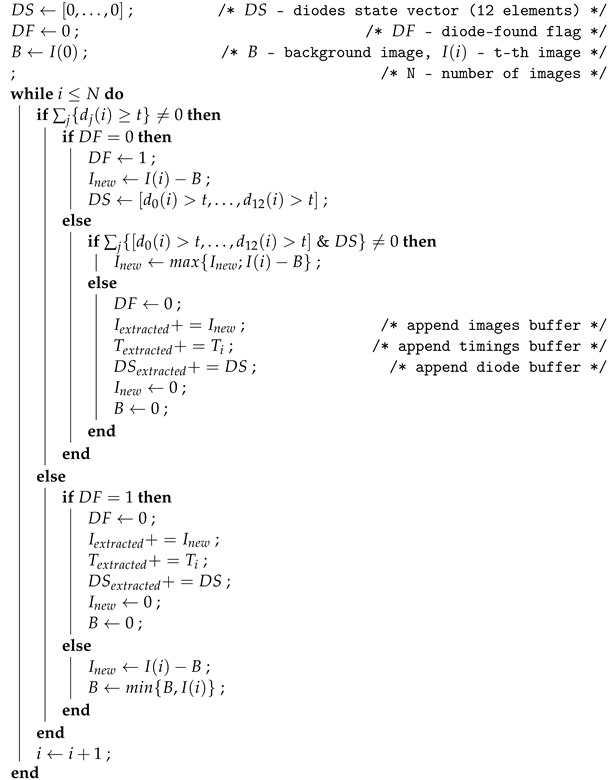


In the process of calibrating the device, we decide on the position of the so-called detection pixels, which allows us to detect the state (on or off) of the 7-segment LEDs. Due to the stable fixing of the camera to the modules, once selected, the detector pixels remain unchanged. The illumination level of the *j*th detector pixel in the *i*th image is defined in the algorithm as $d_{j}\left(i\right)$. The threshold of the detectors is also chosen. The brightness of the 7-segment LEDs in the image results in saturation when the LED is on throughout the exposure. In contrast, when turned off, the brightness level is almost zero. To detect the LED diode even if it was turned off for a small percentage of the exposure time, a detection level of 20 ADU was chosen experimentally (saturation occurs at a level of 255 ADU; ADU stands for Analog-to-Digital Units).

There are two details worth noting in the solution used. First, during image extraction, we use the “maximum” operator, which ensures that the registration will correspond to the full illumination of the IR diode during the exposure time. Second, we also apply background (*B* in the algorithm) subtraction so that the varying side illumination, which could potentially reach the device, is virtually eliminated.

**Figure 5:**
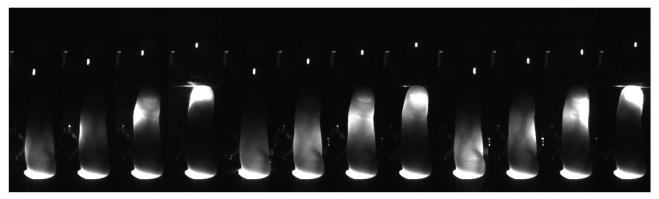
Sample combined series of extracted images.

**Figure 6:**
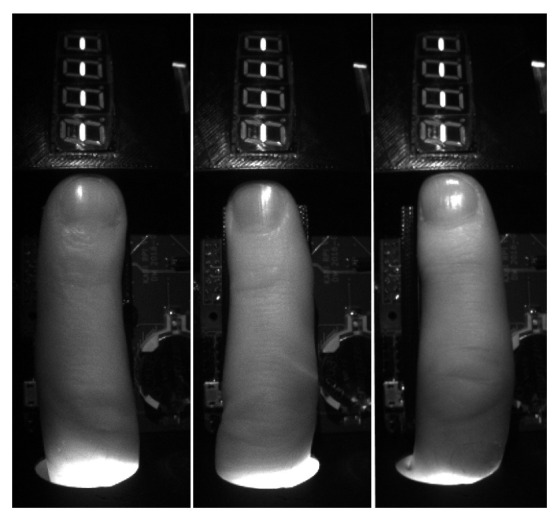
Sample images with front light illumination on (three individuals).

The result of the algorithm is a set of images in buffer $I_{extracted}$, a corresponding buffer of acquisition moments $T_{extracted}$, and a buffer of detected diodes state $DS_{extracted}$. A sample set of images obtained from a sample individual for all 12 back-light NIR diodes is presented in Figure 5. The intensity scale in Figure 5 was adjusted to show structures in all images; the white regions visible within nails are not saturated in the original, raw images. In Figure 6, we present examples of images obtained with front light illumination turned on ($DS_{extracted}=000011110000$) for three different persons.

The type of image sensor we used—the rolling shutter CMOS—is probably the most frequent sensor utilized for both scientific and commercial purposed. However, it should be clearly pointed out that the other more sophisticated image sensors—the frame transfer CCD and the global shutter CMOS—will not need such a specific approach to data selection. They are virtually frozen and insensitive to light during the readout. Still, with a rolling shutter camera we show that post factum image extraction is also possible.

In free-run mode, the ASI ZWO178MM camera achieves ∼130 frames per second (FPS) from which, after post-processing, we retrieve approximately 33 useful frames each second. For comparison, if a camera runs individual shots and the synchronization is performed in real time, only a few frames per second are possible. This is due to the initialization process run each time a single snapshot is requested, while in free-run mode the initialization is performed only once at the beginning of acquisition.

**Figure 7:**
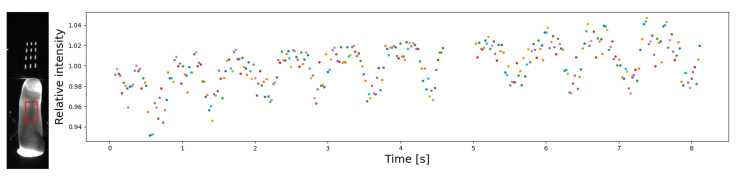
Sample pulse wave as obtained from combining intensity measurements for various NIR diodes. The image on the left shows a summed exposure with a red rectangle indicating the intensity measurement patch. Points colors correspond to the measurements with various NIR diodes.

We tested whether it was possible to identify the pulse wave in the data coming from our device. A rectangular, arbitrary taken region was defined within the finger area, and the average brightness for each image was determined. The average brightness measurements were then normalised individually for each NIR diode so that each extracted signal had an average of 1. The result of combining all signals is given in Figure 7. The pulse wave can be easily seen although the amplitude in this case does not exceeds a few percent. This shows high sensitivity of the presented acquisition device. The ability to detect the pulse wave in our instrument may be important as it safeguards against potential attacks using an artificial finger model.

## 3. Preliminary Results

Sample data were recorded for five people. The index finger and the middle finger of both hands were selected. Each finger was measured in 1000 frames so that the total measurement time was about 7.5 s. Both the raw data and the effect of the post-processing performed by our algorithm (directories with the extension “_processed”) are made available on Zenodo at https://doi.org/10.5281/zenodo.7214386 (accessed on 9 February 2023).

The naming of the raw files corresponds to the second of measurement counting from the start of recording. Processed data have a nomenclature corresponding to the number of the attached NIR diode. For example, the file “D01_0.51.png” indicates that diode number 1 was on and the image was taken at 0.51 s. The frame corresponding to the lighting of the external illumination is labeled with “DEXT”. The full dataset was made available as a zipped archive of about 10 GB.

We also collected a different dataset for comparison of our multi-image approach with the single-image, traditional method. We collected short series of images (up to three cycles of diode pattern sweep, less than a second) and acquired additional image with all diodes on at the end of a cycle. This was repeated 10–15 time for each of 12 persons, each time putting the finger into the machine differently. Then, on such an examination set, we used one of the current state-of-the-art machine learning algorithm—ResNET [48]—with its simplest implementation ResNET18. We had to slightly modify the first convolution layer so that it accepts either 1 or 12 input images, which corresponds to the classic single-image (full illumination) or the 12-images (images for different didoes) classification. The remaining interiors of the network remained unchanged compared with the original ResNET18 structure to preserve the capabilities of such a deep neural network (DNN) in the two types of input data.

To train the DNN, we used the *PyTorch* Python library with a Stochastic Gradient Descent (SGD) optimization [49] and a cross-entropy loss function. These are frequently used methods utilized in similar image classification tasks [50]. As a training set, we selected images fro m2 (random) of the 10–15 trials. This was dictated by the small number of subjects (only 12) and corresponding simplicity of the classification task for DNN of images. If a frequently used fraction of 80% of the dataset was utilized as a training set, the results of both approaches would be very close to 100%, thus indistinguishable. Therefore, to compensate for the limited number of subjects, we decided to make the task harder by limiting the amount of data available in the training set, and simultaneously, we left a much larger dataset for testing the algorithms. The training process utilized 30 epochs which appeared to be sufficient to stabilize the final accuracy on the validation set. The training was repeated 500 times, giving 500 final models for each of the two approaches.

**Figure 8:**
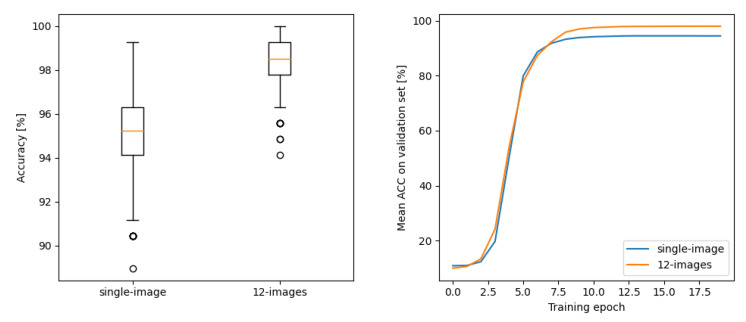
Accuracy results from experiments with ResNET classifier. On the left: spread of accuracy for 500 models trained either with a single- or multiple-image input; on the right: the average accuracy achieved by models across the training epochs.

The results of our experiment are presented in Figure 8. The plot on the left side shows the spread of accuracy on the validation set of 500 networks trained either using single-image input or 12 images. A clear superiority of our multi-diode approach can be observed. The averaged accuracy achieved by the networks in each consecutive epoch of a training is given on the plot on the right side. Until 5–6 epochs, both types of input data seem not to differ. However after six epochs, the networks trained with multiple input images achieved a few percent better results, approaching 100%. It should be noted that for verification device/technique, the difference between 95% and 99% is huge. The number false positive decisions, which is here the the most critical quantity, is reduced five times.

## 4. Conclusions

Personal identification using finger vasculature (FV) is currently an intensively developed technical topic. While most of the research work focuses on algorithms that classify images to make an identification decision, there are a small number of emerging devices that introduce a new quality in the biometric features collected.

In this paper, we present a new device that allows the capture of multiple biometric features associated with human finger imaging. Unlike existing solutions, it is possible to collect data in the form of images under selective illumination, both spatially and in terms of light wavelength. Our instrument allows simultaneous recording of the pulse waveform as well as enabling texture analysis of the top layer of skin.

The device is additionally simple to build and relatively inexpensive. The cost of the instrument, including an Intel NUC computer, is well within USD 1000 (cost estimation made on 16 January 2023). It has no moving parts and can, in our opinion, be successfully brought to a reliable commercial solution. The source codes, technical drawings, and the PCD design of the device can be made available for interested readers upon a reasonable request.

Further research work is envisaged to collect more data from many individuals and to make the databases available as a public resource, on which it would be possible to train and compare image processing solutions for personal identification purposes.

## Data Availability

The sample data from our instrument are available via Zeonodo under the link https://zenodo.org/record/7214386, accessed on 6 February 2023. The source codes, technical drawings, and the PCD design of the device can be made available for interested readers upon a reasonable request.
